# A spatiotemporal steroidogenic regulatory network in human fetal adrenal glands and gonads

**DOI:** 10.3389/fendo.2022.1036517

**Published:** 2022-11-17

**Authors:** Yifu Wang, Bingqian Guo, Yajie Guo, Nana Qi, Yufang Lv, Yu Ye, Yan Huang, Xinyang Long, Hongfei Chen, Cheng Su, Liying Zhang, Qingyun Zhang, Minxi Li, Jinling Liao, Yunkun Yan, Xingning Mao, Yanyu Zeng, Jinghang Jiang, Zhongyuan Chen, Yi Guo, Shuai Gao, Jiwen Cheng, Yonghua Jiang, Zengnan Mo

**Affiliations:** ^1^ Center for Genomic and Personalized Medicine, Guangxi Medical University, Nanning, Guangxi, China; ^2^ Guangxi Collaborative Innovation Center for Genomic and Personalized Medicine, Guangxi Medical University, Nanning, Guangxi, China; ^3^ Guangxi Key Laboratory for Genomic and Personalized Medicine, Guangxi Medical University, Guangxi, China; ^4^ Institute of Urology and Nephrology, The First Affiliated Hospital of Guangxi Medical University, Nanning, Guangxi, China; ^5^ Department of Urology, The First Affiliated Hospital of Guangxi Medical University, Nanning, Guangxi, China; ^6^ Guangxi Collaborative Innovation Center for Biomedicine (Guangxi-Association of Southeast Asian Nations (ASEAN) Collaborative Innovation Center for Major Disease Prevention and Treatment), Guangxi Medical University, Nanning, Guangxi, China; ^7^ Department of Emergency, The Second Affiliated Hospital of Guangxi Medical University, Nanning, Guangxi, China; ^8^ Department of Urology, The Second Affiliated Hospital of Guangxi Medical University, Nanning, Guangxi, China; ^9^ Department of Obstetrics, The Second Affiliated Hospital of Guangxi Medical University, Nanning, Guangxi, China; ^10^ School of Public Health of Guangxi Medical University, Guangxi Medical University, Nanning, Guangxi, China; ^11^ Department of Gynecology, The Second Affiliated Hospital of Guangxi Medical University, Nanning, Guangxi, China; ^12^ Department of Urology, The Affiliated Tumor Hospital of Guangxi Medical University, Nanning, Guangxi, China; ^13^ Department of Gynecology and Obstetrics, The First Affiliated Hospital of Guangxi Medical University, Nanning, Guangxi, China; ^14^ College of Animal Science and Technology, China Agricultural University, Beijing, China; ^15^ Life Sciences Institute, Guangxi Medical University, Nanning, Guangxi, China

**Keywords:** adrenal glands, Sc-RNA sequencing, steroidogenic regulation network, sexual differentiation, spatial transcriptomics

## Abstract

Human fetal adrenal glands produce substantial amounts of dehydroepiandrosterone (DHEA), which is one of the most important precursors of sex hormones. However, the underlying biological mechanism remains largely unknown. Herein, we sequenced human fetal adrenal glands and gonads from 7 to 14 gestational weeks (GW) *via* 10× Genomics single-cell transcriptome techniques, reconstructed their location information by spatial transcriptomics. Relative to gonads, adrenal glands begin to synthesize steroids early. The coordination among steroidogenic cells and multiple non-steroidogenic cells promotes adrenal cortex construction and steroid synthesis. Notably, during the window of sexual differentiation (8–12 GW), key enzyme gene expression shifts to accelerate DHEA synthesis in males and cortisol synthesis in females. Our research highlights the robustness of the action of fetal adrenal glands on gonads to modify the process of sexual differentiation.

## Introduction

The human fetal adrenal gland is a large endocrine organ consisting of a definitive zone (DZ), a fetal zone (FZ, approximately 80-90%) (1), and a transition zone [TZ, after 14 gestational weeks (GW)] that produces large amounts of dehydroepiandrosterone (DHEA) and cortisol (1–3). For sex hormones, DHEA is the most prevalent precursor (2, 4, 5). DHEA is certainly the primary source of placental estradiol (E2), which is essential for regulating the development and maturation of the fetus and the maternal-fetal interface (2, 6). In the testis or ovary, DHEA is processed into a distinct subtype of testosterone or estrogen, which promotes sexual differentiation in organs (2, 3, 7).

Human fetal gonads were “bipotential” (8) and their development was hormone dose-dependent until the 8 GW gonads began regulating the sexual differentiation of the external genital organs (5, 9). The descent of the testis and the formation of the external genitalia in males are supported by the production of enough testosterone in the testis (5, 10) whereas in females, the ovary suppresses the production of excess androgens, which might result in masculinization (11). Extensive pieces of evidence have demonstrated that dysfunction of fetal adrenal glands contributes to reproduction-related diseases, including polycystic ovary syndrome (4, 12), impaired spermatogenesis, and testicular dysfunction (13). The malfunction of adrenal glands is caused by mutations in several genes, such as CYP21A2 (Cytochrome P450 Family 21 Subfamily A Member 2), CYP11B1 (Cytochrome P450 Family 11 Subfamily B Member 1), and POR (Cytochrome P450 Oxidoreductase), and when adrenal dysfunction occurs before the window of sexual differentiation, the malfunction may lead to severe virilization or feminization (7, 14). Based on the occurrence of DHEA circulation in fetal adrenal glands and gonads, we hypothesized that adrenal glands and the regulatory network for steroidogenesis between adrenal glands and gonads may play a critical role in the early stages of sexual development during the window of sexual differentiation.

However, it is challenging to investigate the functions of human fetal adrenal glands for two reasons. First, compared to other mammalian species, humans and nonhuman primates have different origins for circulating DHEA and androgens generated from the adrenal gland (2, 15). Second, adrenal glands are a complex structure with various cellular component organs consisting of numerous heterogeneous cell types. The majority of cortical cells are derived from mesoderm, neurocytes originate from the ectoderm (16, 17). The precise composition of tissue structures and functions of fetal adrenal glands are still unclear. Single-cell sequencing studies of the human adrenal glands are rare (18).

Recent advancements in single-cell sequencing technology have made it possible to map out the complex cell biology landscape in tissue (16, 19–22). We can conduct a comprehensive and thorough analysis of the spatial and temporal information inside the organization from the dimension of multi-omics by combining sc-RNA sequencing with spatial transcriptomics (23, 24). In this study, we created an in-depth map of the adrenal glands and gonads of 7-14 GW human fetuses, which provides an unprecedented resource to help with understanding the intricacy of adrenal glands and sheds light on the role of fetal adrenal glands and gonads in modifying the process of sexual differentiation.

## Materials and methods

### Human samples and quality control

Human fetal adrenal and gonad tissues were isolated from aborted fetuses following the elective surgical termination of pregnancy at the Second Affiliated Hospital of Guangxi Medical University, China. Every donor for the study provided informed consent. Gestational age was validated by ultrasound for crown rump length and fetal limb length to obtain more precise information about the age of the fetuses as described previously (25). The sex of the fetus can be determined by photographs of external genitalia or PCR *SRY* (Sex Determining Region Y) gene detection. All applicable institutional and/or national guidelines for the care and use of animals were followed. The study was approved by the ethics committee of the Second Affiliated Hospital of Guangxi Medical University (KY-0096).

### Preparation of cell suspension

Fresh samples of human fetal adrenal glands or gonads were placed in RPMI 1640 medium (C11875500BT, Gibco), which contained 5% fetal bovine serum (FBS; SH30070.03, HyClone) and 1% penicillin–streptomycin (15140-122, Gibco), and quickly transported to the laboratory on ice. Then, the fetal adrenal glands and gonads were separated under a stereomicroscope (Nikon), washed with cold D-PBS (311-425-CL, Wisent) and sliced into approximately 1–2-mm^3^ pieces. The tissues were transferred to digestion solution [0.1 mg/ml Liberase TL (5401020001, Roche) and 1 mg/ml DNase I (10104159001, Roche) in RPMI 1640] at 37°C with gentle shaking throughout (adrenal glands for 20 min and gonads for 30 min), filtered through a 100-μm cell strainer (352360, Falcon), centrifuged and resuspended in 5 ml of 1X red blood cell (RBC) lysis buffer (420301, BioLegend) for 5 min on ice. Then, the cells were washed twice with D-PBS containing 1% FBS, filtered through a 40-μm cell strainer (352340, Falcon), centrifuged and resuspended in DPBS with 1% FBS. The cell number and viability were assessed by Trypan blue (152 50-061, Gibco) staining and counting in a counting chamber (717805, Brand).

### Single-cell RNA-seq library preparation and sequencing

We followed the approach of our previous studies (26, 27). Reverse transcription and library preparation were performed using the 10x Genomics Single Cell v3 Kit following the 10x Genomics protocol. Briefly, we added the single-cell suspension, gel beads and partitioning oil to 10x Genomics Chromium Chip B and ran the Chromium Controller. After water-in-oil generation, samples were transferred into a PCR tube, and reverse transcription was performed using a T100 Thermal Cycler (Bio-Rad). Then, cDNA purification and library preparation were performed in accordance with the user guide. cDNA libraries were sent to Genergy Biotech (Shanghai) and sequenced by NovaSeq 6000 (Illumina).

### Spatial transcriptome library preparation and sequencing

Fetal adrenal gland tissue samples were embedded in optimal cutting temperature compound and stored at −80°C in a sealed container. The RNA integrity number of the tissue sections should be confirmed to be ≥ 7 prior to mounting the tissue sections onto Visium Spatial slides. Cryostat temperature settings of –20°C for the blade and –10°C for the specimen head were recommended. Tissue blocks were cut into 10-μm sections, and the capture areas were processed using the Visium Spatial Gene Expression Kit (10x Genomics) according to the manufacturer’s instructions. The fetal adrenal gland tissue permeabilization condition was optimized using the Visium Spatial Tissue Optimization Kit. Sections were stained with H&E and imaged using a Nikon Eclipse Ti2 microscope, followed by processing for spatial transcriptomics by consulting the Visium Spatial Gene Reagent Guidelines Technical Note (CG000239) for more information. After library construction, an Agilent 2100/LabChip GX Touch was used to detect the fragment length distribution of the library. Additionally, the q-PCR technique was used to accurately quantify the effective concentration of the library, which was > 10 nmol/L. The qualified library was sequenced on an Illumina NovaSeq 6000 platform. The cycling conditions (sequencing base length) were set to 28, 90 and 10 for read 1, read 2 and read 3 (i7 index), respectively.

### PCR

DNA was extracted from tissue samples according to the kit instructions (DN10, Aidlab). Fragments of the exons of *SRY* genes were amplified by CWBIO 2*ES Tap Master Mix (Dye) (CW0690M, CWBIO) with primers (forward 5′-CAGGATAGAGTGAAGCGACC-3′ and reverse 5′-CATAAGAAAGTGAGGGCTGTAAG-3′) in a 50-μl reaction mixture. PCR was performed using the Bio-Rad T100 Thermal Cycler with the following PCR program: 94.0°C for 2 min; 50 cycles of 94.0°C for 30 sec, 60.0°C for 30 sec, and 72.0°C for 30 sec); and 72.0°C for 2 min.

### Immunofluorescence staining

The following protocols were used for staining of the fetal adrenal glands or gonads: paraffin-embedded, 4-µm-thick formalin-fixed tissue sections were dewaxed in xylene and rehydrated with distilled water. The sections were treated with EDTA at a pH of 8.5 (C1034, Solarbio) to induce epitope retrieval by heating. After the samples were blocked for 30 min in phosphate-buffered saline (PBS, SH30256.01, HyClone) with 2% bovine serum albumin (BSA, 9048-46-8, Sigma), sections were incubated overnight at 4°C with primary antibodies: rabbit anti-human StAR (Catalog # NBP1-33485, RRID: AB_2197666), mouse anti-human HSD3B2 (Catalog # sc-515120, RRID: AB_2721058), goat anti-human CYP17A1 (Catalog # NB100-2842, RRID: AB_789512), rabbit anti-human NOV (Catalog # PA5-27893, RRID: AB_2545369), mouse anti-human CYP17A1 (Catalog # sc-374244, RRID: AB_10988393), goat anti-human MC2R (Catalog # NB100-93419, RRID: AB_1237169), mouse anti-human Chromogranin A (LK2H10) (Catalog # MA5-13096, RRID: AB_10987033), mouse anti-human AKR1C2 (Catalog # NBP2-79775, RRID: AB_2910089), goat anti-human SRD5A1 (Catalog # NB100-1491, RRID: AB_2255212), rabbit anti-human CD5L (Catalog # NBP1-76700, RRID: AB_11020303), mouse anti-human CD68 (Catalog # 14-0688-82, RRID: AB_11151139), rabbit anti-human CHGA (Catalog # 10529-1-AP, RRID: AB_2081122), mouse anti-human NPY (Catalog # ABS 028-08-02, RRID: AB_1077304), rat anti-human SST (Catalog # MA5-16987, RRID: AB_2538460), rabbit anti-human POMC (Catalog # bs-6942R, RRID: AB_10857893), rabbit anti-human TGFb1 (Catalog # bs-0086R, RRID: AB_10856457), rabbit anti-human AGT (Catalog # NBP1-30027SS, RRID: AB_1968450), mouse anti-human ACTA2 (Catalog # MA1-06110, RRID: AB_557419), rabbit anti-human AMH (Catalog # PA5-35851, RRID: AB_2553161), and mouse anti-human DSC2 (Catalog # 32-6200, RRID: AB_2533090). The sections were washed three times with PBS for 5 min each and then incubated with Alexa Fluor 647-conjugated donkey anti-goat IgG (Catalog # ab150135, RRID: AB_2687955), Alexa Fluor 594-conjugated donkey anti-mouse IgG (Catalog # ab150112, RRID: AB_2813898), Alexa Fluor 488-conjugated donkey anti-rabbit IgG (Catalog # ab150061, RRID: AB_2571722), or Alexa Fluor 647-conjugated donkey anti-rat IgG antibodies (Catalog # ab150155, RRID: AB_2813835) for 1 h at room temperature. The slides were washed three times in PBS for 5 min each, and the nuclei were then stained with DAPI (4083S, CST). Fluorescence images were captured using a laser scanning confocal microscope (TCS SP8, Laika Microscope System Shanghai Trading Co., Ltd.) and then processed with ImageJ software (NIH). Detailed antibody information can be found in **Table S10**.

### Flow cytometry analysis and cell sorting

The cells were suspended in PBS (containing 1% BSA) and then incubated with antibody (CD68, 1:100, 14-0688-82), at 4°C for 30 min. CD68 was used to sort macrophages (19), which were then washed twice with PBS containing 1% BSA. Then, the cells were incubated with Alexa Fluor 488-conjugated donkey anti-mouse IgG (1:500, ab150117, RRID: AB_2688012) at 4°C in the dark for 30 min and washed twice with PBS containing 1% BSA. The cells were then resuspended in PBS containing 1% BSA for FACS. All samples were loaded on a BD FACS Melody for flow cytometry cell sorting. After sorting, CD68-positive cells were used for cell coculture.

Fetal gonadal cells were fixed with fixation buffer (420801, Biolegend) at room temperature in the dark for 20 min and then washed twice with 1X Intracellular Staining Perm Wash Buffer (421002, Biolegend). The cells were incubated with antibody (SST, 1:100, MA5-16987, Invitrogen) at 4°C for 30 min and washed twice with 1X Intracellular Staining Perm Wash Buffer. Then, the cells were incubated with Alexa Fluor 647-conjugated donkey anti-rat IgG antibodies (1:500, ab150155, Abcam) at 4°C in the dark for 30 min, washed twice and resuspended with 1X Intracellular Staining Perm Wash Buffer. All samples were loaded on a BD C6 Plus for flow cytometry analysis.

### Cell culture

First, primary cell populations isolated from fetal adrenal glands were cultured in 6-well plates (3516, Corning) with 2 ml of medium in RPMI 1640 with 10% FBS (SH30070.03, HyClone), 1% penicillin/streptomycin (15140-122, Gibco) and 2 mM L-glutamine (25030081, Gibco) at 37°C in a humidified 5% CO_2_ atmosphere. After 7 days, expanded adherent cells were trypsinized and reseeded in DMEM/F12 supplemented with 10% FBS, 5% horse serum (16050-122, Gibco), 100 μg/ml primocin (ant-pm-1, *In vivo*Gen), 100 ng/ml recombinant human FGF2 (100-18C, PeproTech), and 2 mM L-glutamine (28). Primary cell populations were stained with CYP17A1 and MC2R to confirm that they were steroidogenic cells.

### Cell stimulation and coculture

Macrophages and steroidogenic fetal adrenal cells were cocultured in 24-well Transwell chambers (3422, Corning). Steroidogenic fetal adrenal cells were incubated in the upper chamber at 6×10^4^ cells/well, and macrophages were inoculated in the lower chamber at a density of 4×10^4^ cells/well. The macrophage-free group was used as a control. Basal DHEA was measured in the supernatants of cell culture after 24 h of cultivation. The concentrations of DHEA and T in cell culture supernatants were detected by ELISA.

### ELISA

Quantification of hormones was measured by ELISA with the following kits: DHEA ELISA kit (KJ-0766, Jiangsu Kejing Biological Technology, RRID: AB_2910096) and testosterone ELISA kit (KJ-0779, Jiangsu Kejing Biological Technology, RRID: AB_2910095) according to the manufacturers’ instructions.

### Processing of single-cell RNA-seq data

Raw data were demultiplexed using the mkfastq application (Cell Ranger v3.1.0) to generate Fastq files. Fastq files were then analyzed with the count application (Cell Ranger v3.1.0) using default settings, which perform alignment (using STAR aligner, aligned to the GRCh38 human reference genomic data), filtering and UMI counting. UMI count tables were used for further analysis.

### Visium spatial transcriptomics data processing

Reads were demultiplexed and mapped to the reference genome GRCh38 using Space Ranger software v.1.0.0 (10x Genomics). Count matrices were loaded into Seurat v.3.1.1 for all subsequent data filtering, normalization, filtering, dimensional reduction and visualization. Data normalization was performed on independent tissue sections using the variance-stabilizing transformation method implemented in the SCTransform function in Seurat.

### Cell-type identification and dimensionality reduction

The “Seurat” package (v.3.1.1) (23) was used as the first analytical package. For 10x Genomics data, UMI count tables from both replicates from all fetal adrenal gland and gonad samples were loaded into R using the ‘Read10X’ function, and ‘Seurat’ objects were built from each sample. Each object was filtered and normalized with default settings. Specifically, cells were retained only if they contained > 200 expressed genes and < 4,500 genes and had < 15% reads mapped to the mitochondrial genome. After the cell filtration of each object, we used the ‘merge’ function from “Seurat” to combine the objects into two main objects according to the source of organs, adrenal or gonad, for downstream analysis. Cells were normalized to the total UMI read counts by the ‘NormalizeData’, ‘FindVariableFeatures’ and ‘ScaleData’ functions, guided by tutorial in https://satijalab.org/seurat. Then, the objects were explored with the view of molecular networks to remove the batch effect by the ‘RSCORE’ function from the “RSCORE” package (29). Principal component analysis was performed by the ‘RunPCA’ function from “Seurat”, and the top 35 principal components were selected for UMAP analysis. The UMAP (30) analysis was performed by the ‘RunUMAP’ function from “Seurat”. Similar cells were clustered and detected using the Louvain method for community detection by the ‘FindNeighbors’ function. Discrete clusters from adrenal and gonad data were detected using ‘FindClusters’ and annotated by specific cell markers from the “cellmarker” database (http://bio-bigdata.hrbmu.edu.cn/CellMarker/). The erythrocytes (*HBB*, *ALAS2*) were filtered out by the ‘subset’ function. After removal of erythrocytes, 75,482 adrenal cells and 53,508 gonad cells were retained for downstream analysis.

The screened cells were reclustered using the same analytical parameters. Forty-two discrete clusters from adrenal data and 25 from gonad data were detected. The clusters were annotated on the basis of feature genes. Finally, the clusters were regrouped into the main cell groups by definition and similarity.

### Cell cycle analysis

As in our previous studies (27), cell cycle analysis was performed by the “Seurat” package by using previously defined cell cycle genes. We calculated a ‘cycle score’ for each cell based on the expression of cell cycle genes. Cells were considered aperiodic if the cycle score was less than two; otherwise, they were considered proliferating.

### Gene ontology and KEGG analysis

To detect differences in gene function expression among cell clusters in single-cell data, we generated GO gene sets using datasets from “Seurat” single-cell object conversion. GO enrichment analysis was conducted using the “clusterProfiler” package (V3.12.0) (31). Before analysis, we transferred gene names from ‘symbol’ to ‘entrezid’ according to the “org.Hs.eg.db” package (V3.8.2) (32). Terms with a p value < 0.05 were considered and enriched as significant. Dot plots and gene-concept networks were illustrated using the “enrichplot” package (V1.4.0) (33).

### Developmental pseudotime analysis

Detailed pseudotime for different cell types was performed using the “Dyno” package (v2.10.1) (34) following the guideline settings. The inference methods in Dyno were wrapped within “Docker” containers (available at https://methods.dynverse.org). First, the ‘wrap_expression’ function was used to generate Dyno objects by transforming ‘Seurat’ object counts and normalized expression data. Then, the Dyno objects were preset to a “start_id” as an initial state of the cell by the “add prior information” function. In addition, the “add_grouping” function was used to add the results of the cell definition from ‘Seurat’ before the trajectory analysis. Finally, the “PAGA tree” method (35) was selected for trajectory analysis based on the characteristics of our data. Visualization of trajectory analysis was shown by the ‘plot_dimred’ function. All trajectory analysis models were constructed in the correct biological context.

### Cell–cell interaction network analysis

The network analysis of signal interactions between cells was performed using the “CellChat” package (36). The CellChat data were generated from the previous ‘Seurat’ object content by the ‘data.input’ function. The ‘idents’ in the CellChat data were obtained by the cell labels in Seurat objects. ‘Secreted signaling’ and ‘cell–cell contact’, two models of cell interaction, were pulled out from ‘CellChatDB’ for our follow-up analysis by the ‘CellChatDB.use’ function. In the subsequent interpretation of the “CellChat” analysis results, we considered the biological background and ignored some unreasonable results in the gonad data.

## Results

### Expression programs of cell lineages in fetal adrenal glands

**Figure 1:**
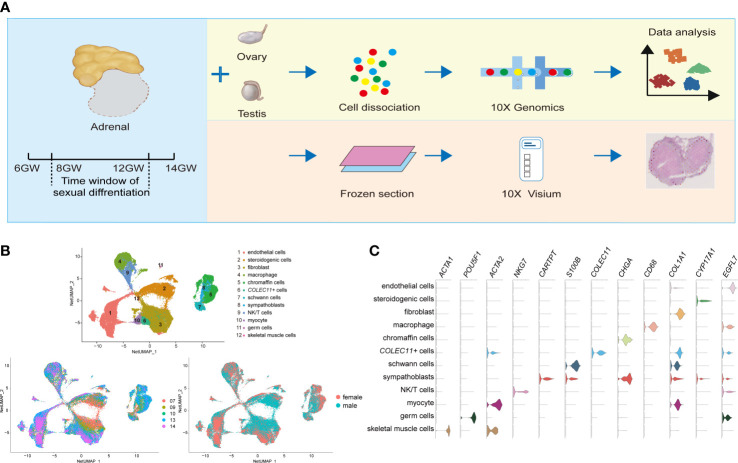
Global patterns of single-cell expression profiles of fetal adrenal glands. **(A)** Experimental schematic; 10 adrenal glands and 8 gonads in 10× Genomics; 2 adrenal glands in the Spatial transcriptome. **(B)** Uniform manifold approximation and projection analysis (UMAP) of the transcriptomes of fetal adrenal cells in 10× Genomics data (n = 75,482). The clusters were identified by marker genes, as shown in **(C)** C. Violin plot overview of the expression of selected marker genes by the fetal adrenal clusters. Detailed cell information and differentially expressed genes can be found in **Table S2**.

To generate a comprehensive transcriptome landscape of cells in fetal adrenal glands and gonads for regulating steroidogenic synthesis spanning the window of sexual differentiation, adrenal gland and gonad (7–14 GW) samples were collected. The samples were digested and subjected to single-cell transcriptome sequencing by using the 10× Genomics system. Frozen sections from 8- to 9-GW fetal adrenal tissues were used for spatial transcriptome analysis. The samples were classified into three stages: before (<8 GW), within (8–12 GW), and after the window of sexual differentiation (>12 GW) (37) (**Figure 1A**). A total of 75,482 adrenal cells and 53,508 gonad cells passed the standard quality control (**Table S1**). To further validate the 10× Genomics results, we also used 1,386 adrenal cells for full-length smart-seq2 transcriptome sequencing (**Figures S1A, B**).

On the basis of 10× Genomics data, uniform manifold approximation and projection analysis (UMAP) revealed 12 main cluster groups (**Figures 1B, C**), including the cells that mainly perform steroidogenic functions that previous studies defined as steroidogenic cells (*CYP17A1*+ (Cytochrome P450 Family 17 Subfamily A Member 1)) (2) as well as other cells, such as neurocytes (*CHGA*+ (Chromogranin A) chromaffin cells, *CARTPT*+ (CART Prepropeptide) sympathoblasts, *S100B*+ (S100 Calcium Binding Protein B) Schwann cells) (16) and immune cells (*CD68*+ macrophages, *NKG7*+ (Natural Killer Cell Granule Protein 7) NK/T cells) (19) (**Figure 1C**; **Table S2**).

As the captured genes increased in Smart-seq2, the expression of the genes encoding steroidogenic enzymes such as *CYP11A1* (Cytochrome P450 Family 11 Subfamily A Member 1), *CYP17A1*, and CYP11B1 (Cytochrome P450 Family 11 Subfamily B Member 1) were found in another types of cell clusters, indicating the involvement of most cells in adrenal steroid metabolism (**Figure S1C**).

### Spatiotemporal transcriptome characteristics of steroidogenic cells

**Figure 2:**
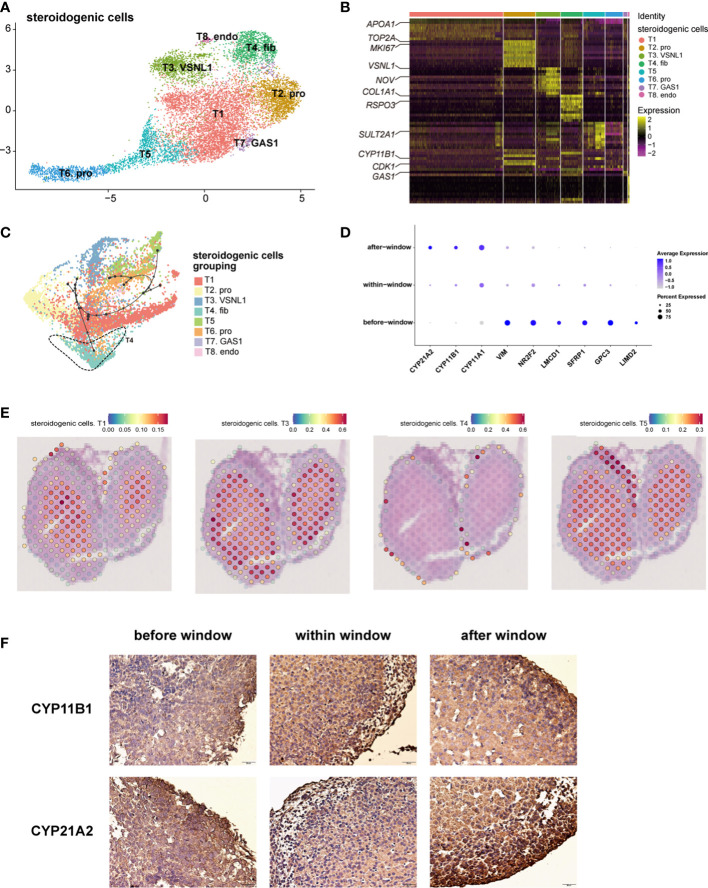
Landscape and characteristics of fetal adrenal gland steroidogenic cells. **(A)** Uniform manifold approximation and projection analysis visualization of steroidogenic cells for 10× Genomics data (n = 10,651). **(B)** Heatmap of the top 10 differentially expressed genes (DEGs) between steroidogenic cell populations (n = 10,651). Color scale: yellow, high expression; purple, low expression. Detailed cell information and DEGs can be found in **Table S3**. **(C)** Differentiation of the trajectory of steroidogenic cells using Dyno. The arrow direction indicates the trajectory of cell differentiation. **(D)** Dot plots of differential gene expression of three-stage steroidogenic cell groups (before, within, and after sexual differentiation). Detailed DEGs of steroidogenic cells between sexes before, within, and after the window of sexual differentiation can be found in **Tables S7-9**. **(E)** Visualization of the spatial transcriptome shows the location of T1 and T3, T4 and T5 steroidogenic cells in 8GW fetal adrenals. **(F)** Immunohistochemical staining of CYP11B1 (up) and CYP21A2 (down) in the fetal adrenal glands spanning the window of sexual differentiation. Scale bar, 20 μm. n = 6.

Steroidogenic cells, classically defined by *CYP17A1*, are a manifestation of adrenal function for steroid biosynthesis (14). We performed an unsupervised analysis of the gene expression profiles of these cells. Eight subtypes of steroidogenic cells were identified (**Figure 2A, B**; **Table S3**). The gene expression heatmap showed that the relative expression levels of the genes known to be associated with steroidogenic metabolism genes (*CYP11B1* and *SULT2A1*(Sulfotransferase Family 2A Member 1) mainly in T1 and T5), proliferation (*MKI67* (Marker of Proliferation Ki-67) and *TOP2A* (DNA Topoisomerase II alpha) in T2), and steroidogenic stem cells (38, 39) (*VSNL1* (Visinin like 1) and *NOV* (also known as *CCN3*, Cellular Communication Network Factor 3) in T3 and *COL1A1* (Collagen Type I alpha 1 chain) and *RSPO3* (R-spondin 3) in T4) varied in the eight subtypes (**Figure 2B**). T2 cells had a high cell cycle score and were defined as proliferative cells (**Figure S2A**). Gene ontology (GO) analysis revealed that chromosome segregation, organelle fission, and nuclear division were enriched in subtype T2 (**Figure S2C**). *NOV* is highly expressed in T3, which is reported to be highly expressed in stem cells located in the subcapsular region and the DZ (2); *SULT2A1* and *CYP11B1* were highly expressed in T1 and T5, which were characterized as the FZ (2) (**Figure 2B**; **Table S3**). Immunostaining showed that cells expressing NOV, MC2R (Melanocortin 2 Receptor) and CYP17A1 were sequentially distributed from the subcapsular region and the DZ to the FZ (**Figure S2B**). The characteristics of T4 cells were similar to those of fibroblasts (**Figure 2B**; **Table S3**). GO analysis revealed that extracellular matrix organization and extracellular structure organization were enriched in this subtype (**Figure S2C**), indicating that T4 may be involved in forming the adrenal zone and capsule.

The spatial transcriptome was used to reproduce the spatial positions of these types of steroidogenic cells. The T1 and T5 steroidogenic cells were located in the FZ of the fetal adrenal glands; the T3 steroidogenic cells were mostly distributed outside of the T1 and T5 steroidogenic cells, which are considered to be the cells located in the DZ (**Figure 2E** and **Figure S2D**). T4 steroidogenic cells, defined herein as stem cells with the potential to differentiate into steroidogenic cells, were distributed only in the peripheral location, which was under the adrenal capsule (**Figure 2E**, **Figure S2D**). In accordance with the classical subcapsular DZ to FZ centripetal differentiation model (2, 38), pseudotime analysis revealed a developmental trajectory of steroidogenic cells from T4 to other subtypes (**Figure 2C**). Consistent with previous sc-RNA seq studies with fetal adrenal (18), with the differentiation and maturity of steroidogenic cells, the steroidogenic-related genes *CYP17A1, CYP11B1* and *CYP21A2* were highly expressed (**Figure S2E**).In summary, the single-cell RNA sequencing data combined with the spatial transcriptome enabled us to support the classical centripetal differentiation model of steroidogenic cells in fetal adrenal glands.

The dot plots showed that along with fetal development, the motility regulator *LIMD2* (LIM Domain Containing 2), transcription factor *NR2F2* (Nuclear Receptor Subfamily 2 Group F Member 2) and mesenchymal marker *VIM* (Vimentin), which are highly expressed in adrenal capsular, pluripotent genes (e.g., *GPC3* (Glypican 3), *SFRP1* (Secreted Frizzled Related Protein 1), and *LMCD1* (LIM And Cysteine Rich Domains 1)), were progressively downregulated. Moreover, the genes encoding steroidogenic enzymes, such as *CYP17A1*, *CYP11B1*, and *CYP21A2*(Cytochrome P450 Family 21 Subfamily A Member 2*)*, were upregulated (**Figure 2D**). Steroidogenic cells with a high expression of *CYP17A1* and *CYP11B1* were enriched in the innermost FZ, while the cells highly expressing *CYP21A2* were highly outside of them (**Figure S2F**). Cells highly expressing *HSD3B2* (Hydroxy-Delta-5-Steroid Dehydrogenase, 3 Beta- And Steroid Delta-Isomerase 2) and *CYP11B2* were in the outermost part of the fetal adrenal glands (**Figure S2F**). *MC2R*, encoding the ACTH receptor protein, is widely expressed in the FZ and TZ (**Figure S2F**). Subsequently, *MC2R* was used as a marker to sort the steroidogenic cells in the inner zone.

### Steroidogenic cells in adrenal glands exhibit distinct expression profiles across different sexes

**Figure 3:**
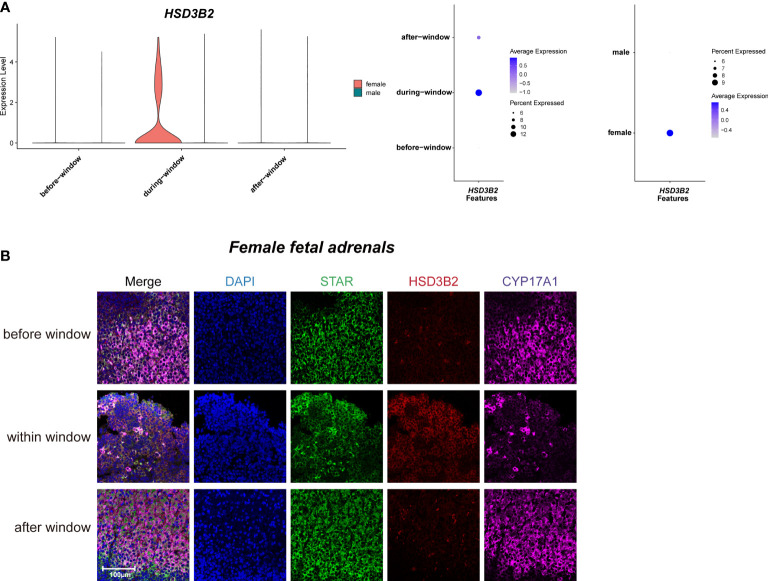
Sexual dimorphic expression patterns of steroidogenic-related genes in steroidogenic cells. **(A)** Violin and dot plots of *HSD3B2* gene expression patterns of female and male fetal adrenal steroidogenic cells spanning the window of sexual differentiation. **(B)** Immunofluorescence staining of StAR (green), HSD3B2 (red), and CYP17A1 (purple) in the female fetal adrenal glands spanning the window of sexual differentiation. Scale bar, 20 μm. n = 3.

As previously reported (3, 40), temporarily high expression of *HSD3B2* was observed within the sexual differentiation window only in females (**Figures 3A, B**). The temporarily elevated *HSD3B2* was thought to increase cortisone production and suppress DHEA synthesis through the competitive consumption of common precursors (3, 40). However, the expression of another key enzyme for producing cortisol, *CYP21A2*, was limited in 10x Genomics data within the sexual differentiation window (**Figure 2D**). To exclude the cause of insufficient sequencing depth, we performed full-length Smart-seq2 analysis to confirm that *CYP21A2* was expression at all stages (**Figure S1D**). Immunohistochemical staining also indicated that *CYP21A2* was expressed at all stages (**Figure 2F**). Besides, the expression of *CYP11B1* was further confirmed by immunohistochemical staining (**Figure 2F**). In conclusion, cortisol production in females can be carried out within the sexual differentiation window.

Taken together, our data revealed the sexual dimorphism expression regulation pattern of steroidogenic enzyme genes in steroidogenic cells, indicating that more cortisol are synthesized in fetal adrenal glands in females. The relationship between the above sex differences in steroidogenic cells and human sexual differentiation deserves further in-depth study.

### The transcriptome characteristics of neurocytes in fetal adrenal glands

**Figure 4:**
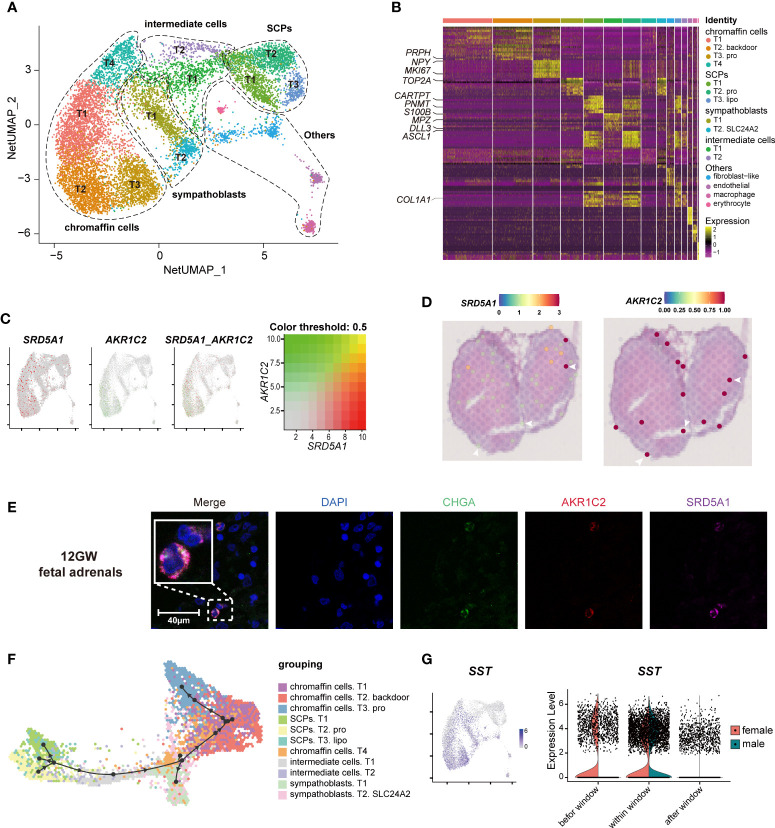
Landscape and characteristics of fetal adrenal gland neurocytes. **(A)** Uniform manifold approximation and projection analysis visualization of adrenal neurocytes for 10× Genomics data (n = 10,812). **(B)** Heatmap of the top 10 differentially expressed genes (DEGs) between adrenal neurocyte populations. Detailed cell information and DEGs can be found in **Table S4**. **(C)** Expression patterns of *SRD5A1* and *AKR1C2* exhibited by feature plot visualization, which are key enzymes of the DHT “backdoor pathway”. A gradient of gray, red, or green indicates low to high expression, and yellow indicates coexpression. **(D)** Visualization of the spatial transcriptome shows the locations with high expression of *SRD5A1* and *AKR1C2* in 8GW fetal adrenal tissues. **(E)** Immunofluorescence staining of SRD5A1 (purple), AKR1C2 (red), and CHGA (green) in fetal adrenal tissues. Scale bar, 20 μm. **(F)** Differentiation of the trajectory of fetal adrenal gland neurocytes using Dyno. The arrow direction indicates the trajectory of cell differentiation. **(G)** Feature plot visualization of *SST* in fetal adrenal neurocyte data, mainly expression in mature neurocytes (chromaffin cells and sympathoblasts). Violin plot of *SST* expression with sex differences.

To explore whether fetal adrenal neurocytes have steroidogenic functions, the neurocyte group was further divided into 15 distinct subtypes, including 4 chromaffin cell subtypes, 2 sympathoblast subtypes, and 3 Schwann cell precursor (SCP) subtypes (**Figure 4A, B**; **Table S4**).

The chromaffin cells expressed the marker genes *CHGA* and *PHOX2B* (Paired Like Homeobox 2B). In addition, T2 chromaffin cells specifically expressed *SRD5A1* (Steroid 5 Alpha-Reductase 1) and *AKR1C2* (Aldo-Keto Reductase Family 1 Member C2), and these cells may synthesize the active androgen dihydrotestosterone (DHT) through a “backdoor pathway” (**Figure 4C-E**) (6, 41). The expression levels were confirmed by immunofluorescence staining (**Figure 4E**).

T2 SCPs expressed the neural progenitor marker genes *S100B* and *SOX10* (SRY-Box Transcription Factor 10), the myelination-associated gene *MPZ*(Myelin Protein Zero), and the cell cycle-associated genes *MKI67* and *TOP2A*, indicating that these cells are actively proliferating cells (**Figure 4B**, **Figure S3A**; **Table S4**). Trajectory analysis revealed that the chromaffin cells and sympathoblasts in humans originated from a cluster of SCPs (20, 42, 43) (**Figure 4F**, **Figure S3B**). This finding is highly consistent with that of previous research in mice (16), and two intermediate cell subtypes were found to serve as a continuous “bridge” between the SCPs and mature neurocytes (**Figure S3B**). These “bridge” cells expressed the SCP marker gene *HOXB9* (Homeobox B9), the functional neurocyte genes *CHGA* and *PHOX2B*, and the transient genes *ASCL1* (Achaete-Scute Family BHLH Transcription Factor 1) and *DLL3* (Delta Like Canonical Notch Ligand 3) (**Figure 4B**, **Figure S3B**; **Table S4**). Along with neurocyte development and maturation, the cell motility gene *ACTC1* (Actin Alpha Cardiac Muscle 1) and the neurodevelopmental genes *ASCL1*, *DLL3*, *GPC3*(Glypican 3), and *HOXB9* (16) were downregulated, whereas the cell mitosis-related genes *HSPA1B* (Heat Shock Protein Family A (Hsp70) Member 1B) and *PROX1* (Prospero Homeobox 1) and the insulin mediator *IRS2* (Insulin Receptor Substrate 2) were upregulated (**Figure S3C**).

In exploring steroid-associated regulators, we found that *SST* (Somatostatin) was highly expressed in both male and female mature neurocytes within the window of sexual differentiation and was also highly expressed before the window in females (**Figure 4G**). Immunofluorescence staining showed that the number of SST+ neurocytes was relatively high during the window of sexual differentiation (**Figures S3D, E**). The spatial transcriptome results suggested that since the adrenal medulla has not been formed at this stage, *SST* expression was dispersed in the fetal adrenal gland (**Figure S3F**). It was previously reported that low concentrations of somatostatin inhibit the production of angiotensin II-stimulated aldosterone in the adrenal zona glomerulosa (44), however, the inhibitory effect by somatostatin in fetal adrenal is unknown. The spatiotemporal expression of *SST* and the sex differences in *SST* reactions in sexual differentiation warrant further research.

### Spatiotemporal transcriptome characteristics of immune cells in fetal adrenal glands

The immune cells were further divided into 16 distinct subtypes (**Figures S4A, B**; **Table S5**), which included 5 macrophage subtypes (*CD68*+ *CD163*+), 2 NK/T-cell subtypes (*NKG7*+ *CD3D*+ (CD3 Delta Subunit Of T-Cell Receptor Complex)), proliferative cell subtypes (*MKI67*+ *TOP2A*+), and yolk sac-derived myeloid-biased progenitors (YSMPs, defined by *CD34*+ *MPO*+ (Myeloperoxidase) *AZU1*+ (Azurocidin 1)) (19). For fetal development, *ACY3* (Aminoacylase 3), *CX3CR1* (C-X3-C Motif Chemokine Receptor 1), and *HMGA2* (High Mobility Group AT-Hook 2), which are associated with chemokines and cell migration, were downregulated, whereas genes such as *RHOB* (Ras Homolog Family Member B), which are involved in mediating apoptosis in immune cells, were upregulated (**Figure S4C**). Macrophages were the most abundant immune cells, and T4 macrophages especially expressed *CD5L* (CD5 Molecule Like) (**Figure S4A, B**). T4 cells were found to be involved in cellular lipid catabolic processes and regulation of plasma lipoprotein particle levels for lipid metabolism (**Figure S4D**). *CD5L* was costained with the macrophage marker *CD68* (**Figure S4E**). In addition, *POMC* (Proopiomelanocortin), which can be cleaved to ACTH, was found to be expressed in macrophages and was confirmed by immunofluorescence (**Figure S4F**). Notably, we found a surge in DHEA secretion by *in vitro* primary fetal adrenal cells cocultured with macrophages, and the crosstalk between macrophages and steroidogenic cells in the adrenal gland needs to be further confirmed (**Figure S4G-H**). Spatial transcriptome analysis revealed that the macrophage markers *CD68* and *CD5L* mentioned above were mainly located in centroids serving as the primary site of FZ (**Figure S4I**).

Macrophages have been found to negatively regulate adrenal proliferation and differentiation *via* the TGFb (Transforming Growth Factor Beta) signaling pathway network (45) (**Figures S5H-J**). These cells may be involved in the phagocytosis of apoptotic steroidogenic cells migrating to the central adrenal glands (2). Our data revealed the transcriptome characteristics of human fetal adrenal macrophages and their spatial location characteristics, inspiring subsequent studies on the mechanisms by which macrophages regulate the development and function of steroidogenic cells.

### Regulatory network of cell–cell interactions in fetal adrenal glands

The complex cell-cell interaction network in fetal adrenal glands provided a microenvironment for fetal adrenal glands to biosynthesize steroid hormones (**Figure S5A**). CellChat analysis showed that the melanocortin signaling pathway acted as an autocrine pathway through the *POMC*–*MC2R* ligand receptor (**Figure S5B**), as verified by spatial transcriptome and immunofluorescence staining (**Figures S5C, D**). NPY (Neuropeptide Y) signaling pathway communication, especially between neurocytes and steroidogenic cells, is associated with cholesterol metabolism (46) (**Figures S3G, H**). The AGT (Angiotensinogen) signaling pathway was found between myocytes and steroidogenic cells through the *AGT-AGTR1* (Angiotensin II Receptor Type 1) ligand receptor (**Figures S5E, F**). *AGT* is an essential component of the renin-angiotensin system cleaved into angiotensin I (Ang I) and II (Ang II) by renin and angiotensin converting enzyme (ACE). In addition to maintaining blood pressure, Ang II can increase the expression of *ACTHR* and enhance the secretion of DHEAS and cortisol in fetal adrenocortical cells (47, 48). The *AGT*-*AGTR1* interaction was demonstrated in the spatial transcriptome (**Figure S5G**).

### Gonadal nonreproductive cells exhibit diverse functions

**Figure 5:**
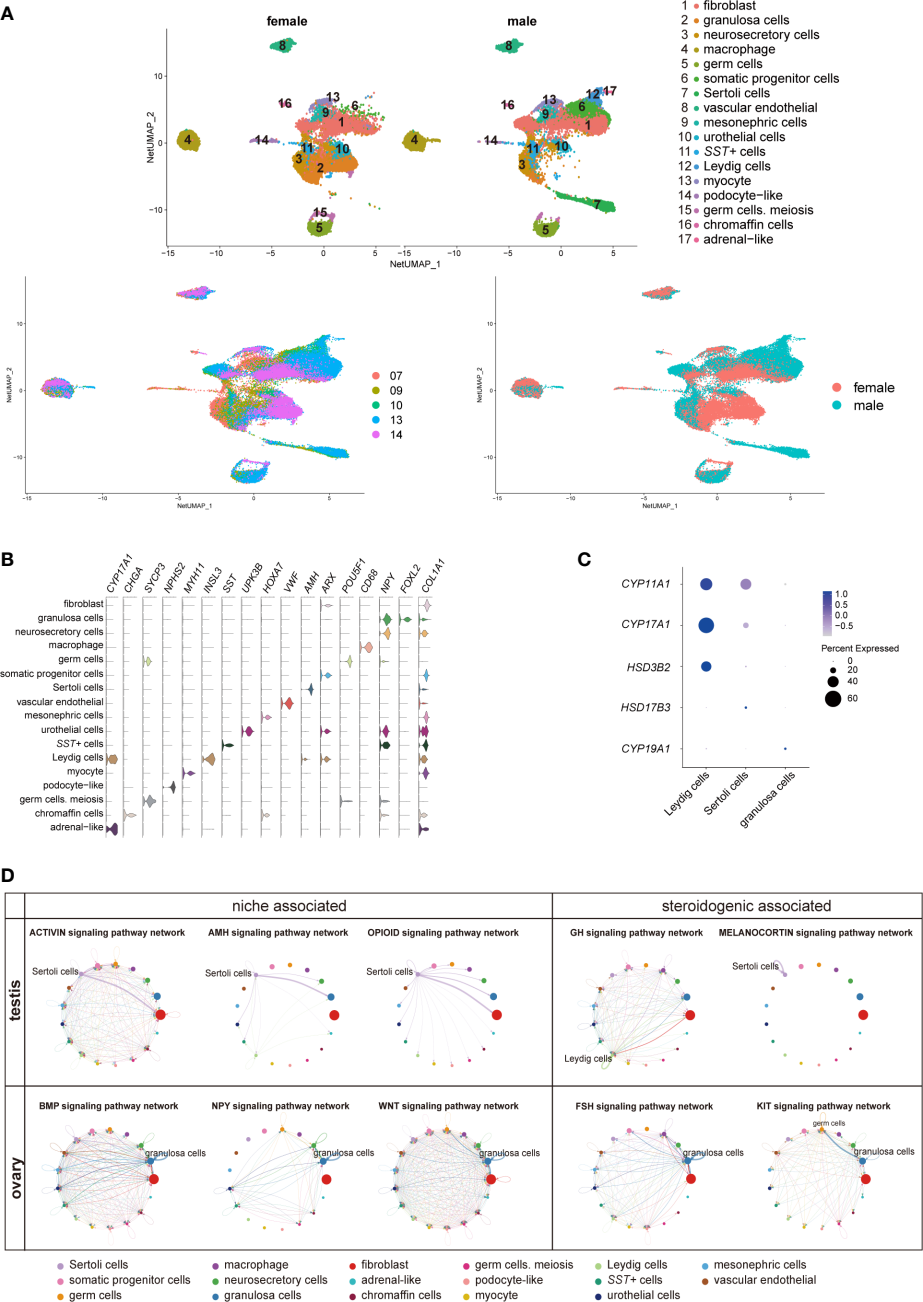
Transcriptomic landscape of human fetal gonads. **(A)** Uniform manifold approximation and projection analysis of the transcriptomes of all-stage fetal gonadal cells split by sex (n = 53,508). **(B)** Violin plot overview of the expression of selected marker genes by gonad clusters. Detailed cell information and differentially expressed genes can be found in **Table S6**. **(C)** Dot plot showing the expression of key enzymes for sex hormone biosynthesis (*CYP11A1*, *CYP17A1*, *HSD3B2*, *HSD17B3*, and *CYP19A1*) in different gonadal somatic cells. **(D)** CellChat analysis showing the cell–cell interaction of gonadal somatic cells in the testis (top). The signal interaction pathway networks are divided into two functions: niche-associated (activin, AMH, and opioid) and steroidogenic-associated (GH and melanocortin). The cell–cell interaction of gonadal somatic cells in the ovary (below). The signal interaction pathway networks are divided into two functions: niche-associated (BMP, NPY, and WNT) and steroidogenic-associated (FSH and KIT). .

To investigate the role of adrenal steroids in gonadal sex hormone synthesis for guiding sexual differentiation, we obtained 17-cell groups from the 10× Genomics gonads data (**Figures 5A-C**; **Table S6**). The groups were subsequently annotated using known gene markers, such as *FOXL2* (Forkhead Box L2) for granulosa cells, *AMH* (Anti-Mullerian Hormone) for Sertoli cells, *INSL3* (Insulin Like 3) for Leydig cells, and *ARX* (Aristaless Related Homeobox) for somatic progenitor cells (8) (**Figure 5B**). *CYP11A1*, *CYP17A1*, and *HSD3B2* were expressed most heavily in Leydig cells, coinciding with their androgen synthesis function (**Figures 5C, D**).

The cell interactions in gonadal somatic cells (Sertoli cells, Leydig cells, and granulosa cells) were explored by CellChat (**Figure 5D**). Sertoli cells can be mediated by the activin, AMH, and opioid signaling pathways to form testicular cords and provide a favorable environment for germ cell survival (21, 49). In addition, Sertoli cells participate in the melanocortin signaling pathway network by coexpressing *POMC*-*MC4R* itself. The role of the melanocortin signaling pathway network may be related to stimulating and promoting the response of Sertoli cells to sex steroid biosynthesis (50) (**Figure 5D**). Leydig cells were found to be regulated by GH (Growth hormone) signaling pathways to induce steroidogenesis (**Figure 5D**) (51). However, GH signaling pathways can cause feminization to some extent (52). The granulosa cell interaction signaling pathway is mainly involved in oogenesis and folliculogenesis in fetal ovaries (21, 53). The WNT signaling pathway promotes granulosa cell differentiation and inhibits apoptosis; the KIT and BMP signaling pathways interact with germ cells to promote oogenesis and folliculogenesis and protect preantral follicles from apoptosis (8, 53) (**Figure 5D**). As a result of immature ovarian granulosa cell development in this period, the function of ovarian steroidogenic regulation by the KIT and FSH signaling pathways may not be reflected (**Figure 5C, D**).

### Steroid enzyme pattern in gonads

Interestingly, *SST* in gonads was specifically expressed in a cluster of cells (**Figures 5A, B**, **S6A**). *SST* can regulate gonadotropins, FSH and LH by the BMP signaling pathway, inhibiting the levels of sex steroids in the ovary (54). The number of *SST*+ cells in males was approximately twice as high as that in females during the window of sexual differentiation (**Figure S6B**). They also showed sexual differences similar to those previously mentioned in adrenal neurocytes (**Figure 4G**). These sexual disparities were confirmed by FACS and immunofluorescence staining (**Figures S6C, D**). GO analysis identified gland development, reproductive structure development, reproductive system development, and mesenchyme development (**Figure S6E**). CellChat analysis indicated that the SST+ cells were in contact with Sertoli cells by desmosomes (**Figure S6F**), which was confirmed by immunofluorescence staining and showed that these two types of cells adhered closely to DSC2 (Desmocollin 2) (**Figure S6G**). The function of SST in steroid hormone metabolism warrants further research.

To describe the potential steroidogenesis network of human fetal adrenal glands and gonads, the results of this research can be summarized as follows: adrenal glands begin to express steroidogenic enzyme genes at approximately 7 GW that produce steroid hormones much earlier than the testis. The steroidogenic enzyme gene expression patterns of the testis support that the testis can *de novo* synthesize testosterone or utilize DHEA from the adrenal gland. An *HSD3B2* expression peak was observed in females at 10 GW. Adrenal glands may synthesize small amounts of DHT. Nevertheless, the expression of steroidogenic enzymes in ovaries was limited until 14 weeks (**Figure S7**).

## Discussion

Temporarily elevated *HSD3B2* was thought to consume pregnenolone by synthesizing cortisol and then reducing DHEA output, thereby avoiding the masculinization of women (3, 40). In our data, *HSD3B2* was highly expressed in females, while *CYP21A2* was limited during the window. High *CYP21A2* expression was not observed until 14 GW. Since *CYP21A2* is one of the key enzymes of cortisol synthesis (3, 40), we conducted immunohistochemical staining to validate that CYP21A2 is expressed at all stages so that the anti-masculinization mechanism exists in females. Surprisingly, *SST* expression was observed to be higher in males not only in adrenal glands but also in gonads during the window of sexual differentiation. *SST*-encoded somatostatin is an inhibitor of growth hormone and inhibits the secretion of other hormones, such as ACTH and GnRH (55–57). We speculate that *SST* expression is probably involved in steroid synthesis in adrenal glands and gonads and may be highly correlated with fetal sexual differentiation. The underlying molecular mechanisms of the sex differences of *HSD3B2* and *SST* require intensive study.

A study in mice reported that the cellular origin from Schwann cell precursors is the stem cell of adrenal medulla cells, which in turn could develop into chromaffin cells or sympathoblasts. A group of intermediate cells that express *Htr3a*+ (5-Hydroxytryptamine Receptor 3A) and *Ascl1*+, which are transient intermediate cells, are called “bridge cells” (16). Notably, consistent with a recent study (43, 58), our research identified human correlates of the four medullary cell types, such as a group of *ASCL1*+ intermediate cells that act as a bridge between SCP and different chromaffin cells and sympathoblasts in human fetal adrenal glands.

Notably, a cluster of *SRD5A1+* and *AKR1C2+* chromaffin cells was found in fetal adrenal glands. These cells may synthesize active DHT by converting An (androsterone) through a process called the androgen synthesis “backdoor” pathway, which corresponds to the conversion of testosterone to DHT *via* the “classic” pathway (6, 41, 59). DHT is considered to be a highly bioactive androgen and reportedly promotes the development and maturation of the nervous system (60, 61). Thus, we surmise that DHT synthesis may promote the maturation of adrenal neurocytes.

A group of *CD5L*+ macrophages was found in fetal adrenal glands, which reportedly mediate lipid biosynthesis in Th17 cells (62). A primary adrenal cell culture test showed a sharp increase in DHEA synthesis in a coculture with macrophages. Whether there is an unknown steroidogenic biosynthesis regulatory mechanism in macrophages deserves further research.

Our study of steroidogenesis in human fetal adrenal glands and gonads during sexual differentiation in a single-cell solution not only confirmed past results but also identified novel cell populations. In summary, these findings can be linked together to reveal a spatiotemporal regulatory network, highlighting spatiotemporal differences in the steroidogenic regulatory network in adrenal glands and gonads, which we propose as a “signal and fuel” phenomenon. *SRY* gene expression is a signal of male differentiation, whereas DHEA synthesis in adrenal glands is the fuel that promotes correct male fetal differentiation. Our analyses provide novel insights into cell crosstalk during sexual differentiation in human fetal adrenal glands and gonads. This study will deepen our understanding of the complex regulatory mechanism of human fetal sexual differentiation.

## Data availability statement

Processed and raw human 10x Genomics data are available *via* the Gene Expression Omnibus (GEO) (GEO: GSE167860).

## Ethics statement

The studies involving human participants were reviewed and approved by The ethics committee of the Second Affiliated Hospital of Guangxi Medical University. The patients/participants provided their written informed consent to participate in this study.

## Author contributions

YW, YJ and ZM contributed to the study design. BG, NQ, YaG and YL mainly performed cell suspensions and 10x single-cell RNA-seq library preparation. YaG, NQ and ZC performed the PCR analysis. BG, YiG, YW and NQ performed immunofluorescence staining and confocal imaging. YiG performed the cell stimulation, coculture and hormone quantification in vitro experiments. BG together with NQ performed the flow cytometry analysis and cell sorting. YW, ZC, JJ, YYe, CS, and QZ collected fetal samples and patient information. YH, HC, LZ, ML and JC provided clinical technical guidance. YW, YYa, XM contributed to the bioinformatics analyses. YW and XM contributed to the manuscript preparation. ZM conceived and directed the study, obtained funding, and revised the manuscript. All authors contributed to the article and approved the submitted version

## Funding

This was supported by grants from the National Key Research and Development Program of China (2017YFC0908000), the Natural Key Research and Development Project (2020YFA0113200), the Major Project of Guangxi Innovation Driven (AA18118016), the Guangxi Key Laboratory for Genomic and Personalized Medicine, the National Natural Science Foundation of China (81770759, 82060145, 31970814), the Natural Science Foundation of Guangxi Zhuang Autonomous Region (2021JJA140912), and the Advanced Innovation Teams and Xinghu Scholars Program of Guangxi Medical University.

## Acknowledgments

We thank the donors for participating in this study. We thank The Second Affiliated of Guangxi Medical University for supporting the sample collection; H. Mi, and L. Mo from The First Affiliated Hospital of Guangxi Medical University; H. Bai, M. Wei, Z. Liu, D. Peng, Y. Su, and S. Yi from the Gynaecology and Obstetrics Hospital of Guangxi Zhuang Autonomous Region; Q. Meng, C. Huang, Y. Xie, D. Li, and P. Wei from Guangxi Medical University; and X. Sun from The Third Affiliated Hospital of Guangzhou Medical University for their experimental help.

## Conflict of interest

The authors declare that the research was conducted in the absence of any commercial or financial relationships that could be construed as a potential conflict of interest.

## Publisher’s note

All claims expressed in this article are solely those of the authors and do not necessarily represent those of their affiliated organizations, or those of the publisher, the editors and the reviewers. Any product that may be evaluated in this article, or claim that may be made by its manufacturer, is not guaranteed or endorsed by the publisher.
