# Supplementary figures and images for: A spatiotemporal steroidogenic regulatory network in human fetal adrenal glands and gonads
Source: Front Endocrinol (Lausanne). 2022 Nov 17;13:1036517. doi: 10.3389/fendo.2022.1036517 (PMC9713933; doi:10.3389/fendo.2022.1036517)

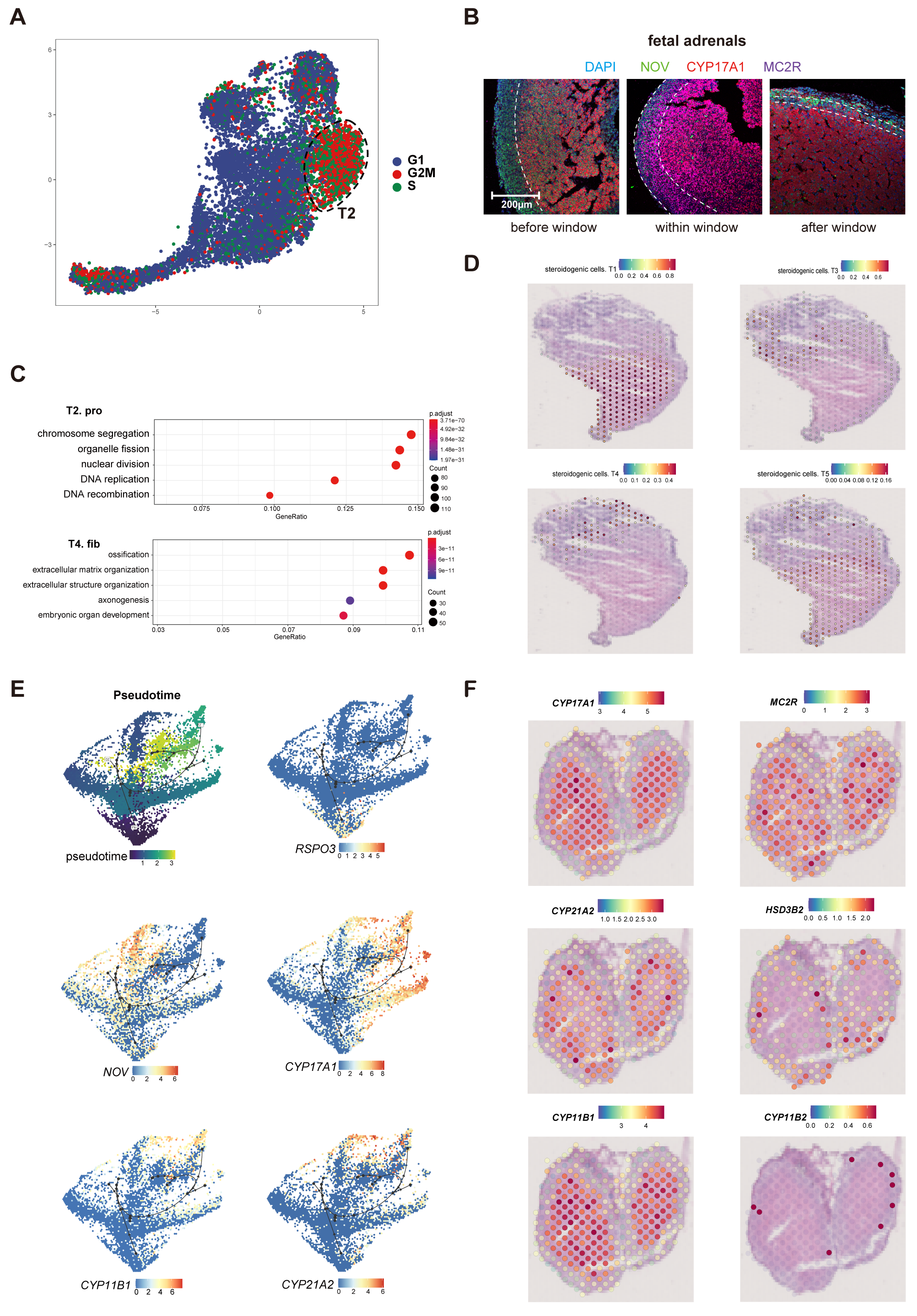

Supplement: Supplementary file 2 [file Image_2.tif]

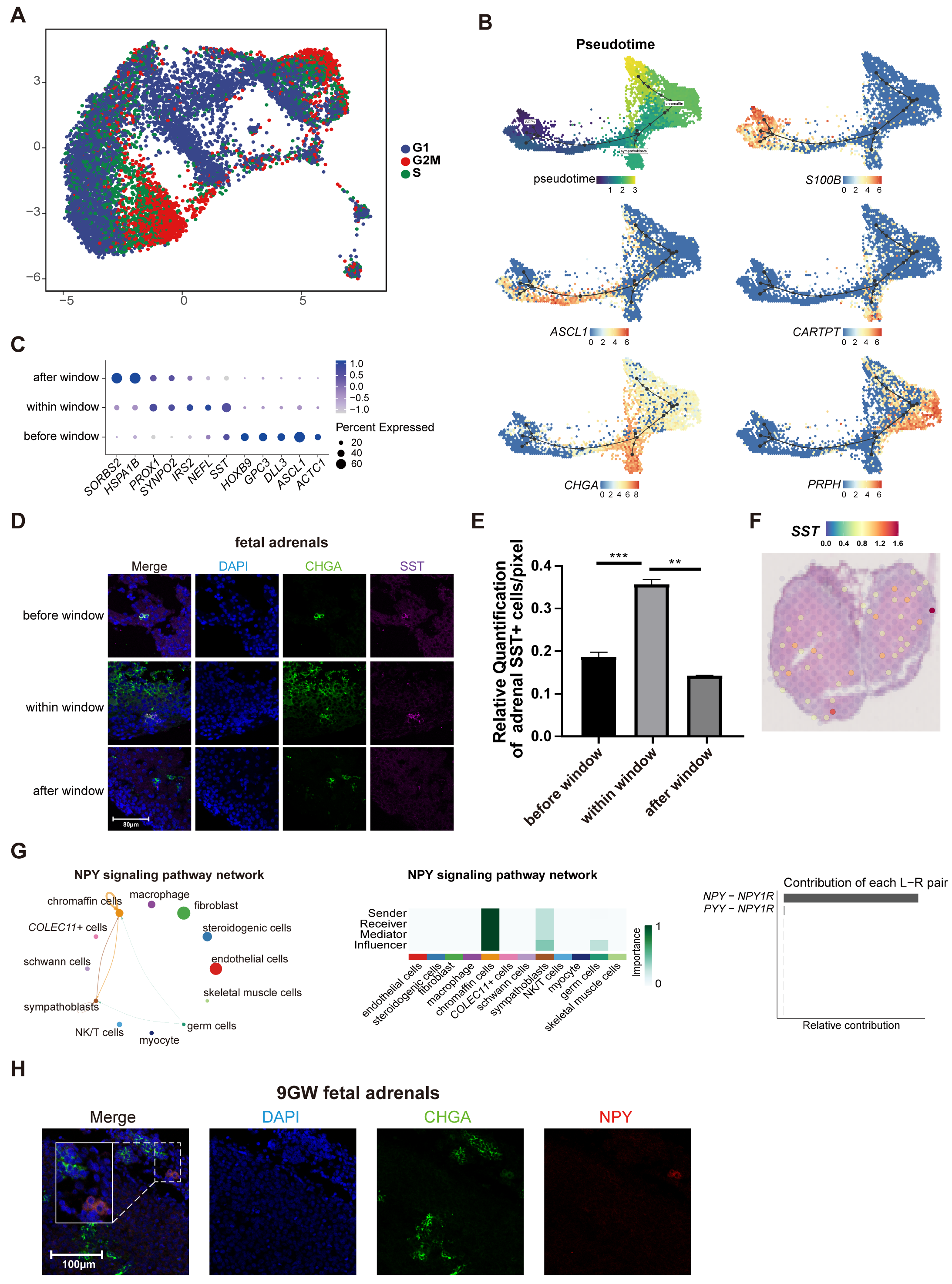

Supplement: Supplementary file 3 [file Image_3.tif]

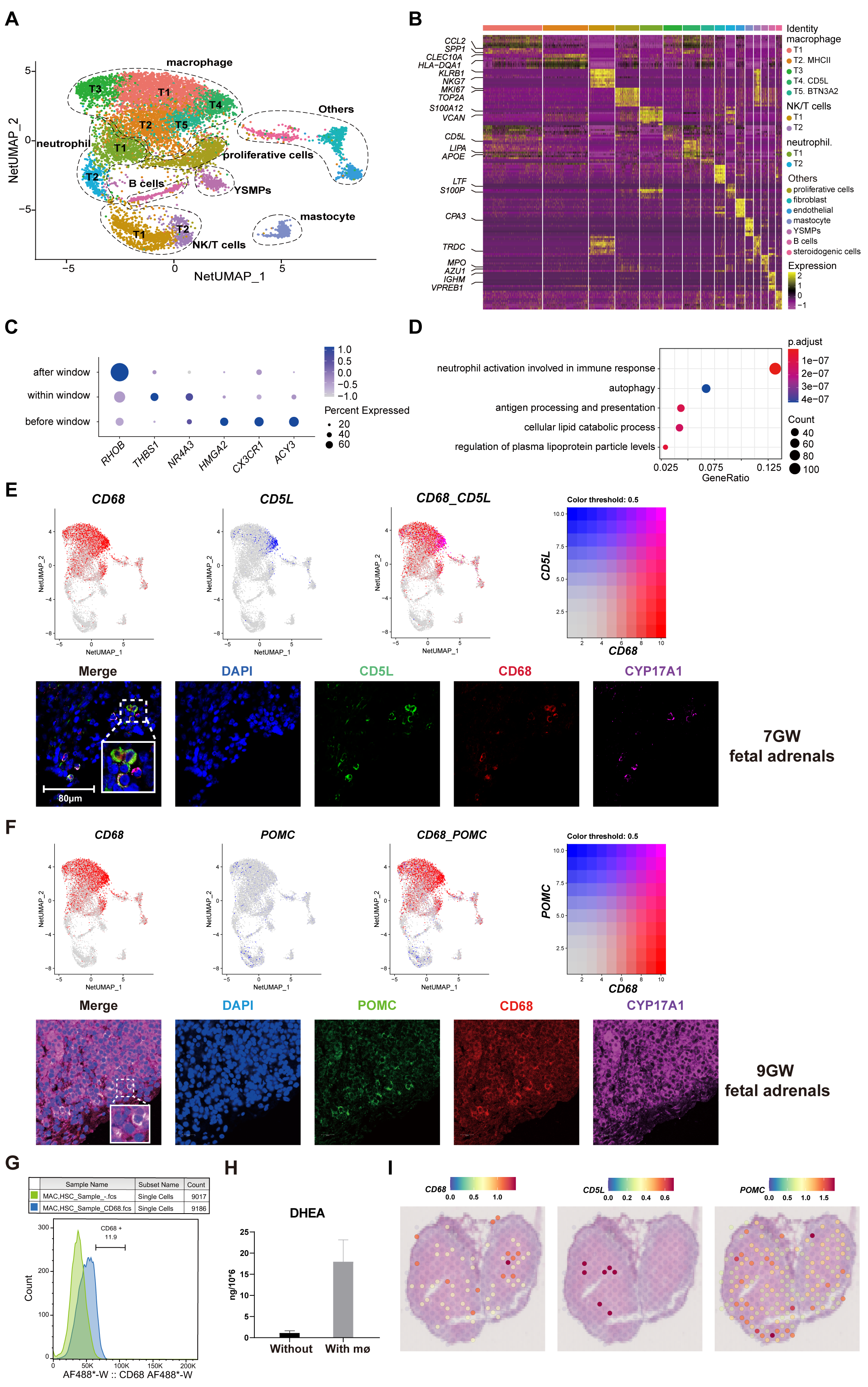

Supplement: Supplementary file 4 [file Image_4.tif]

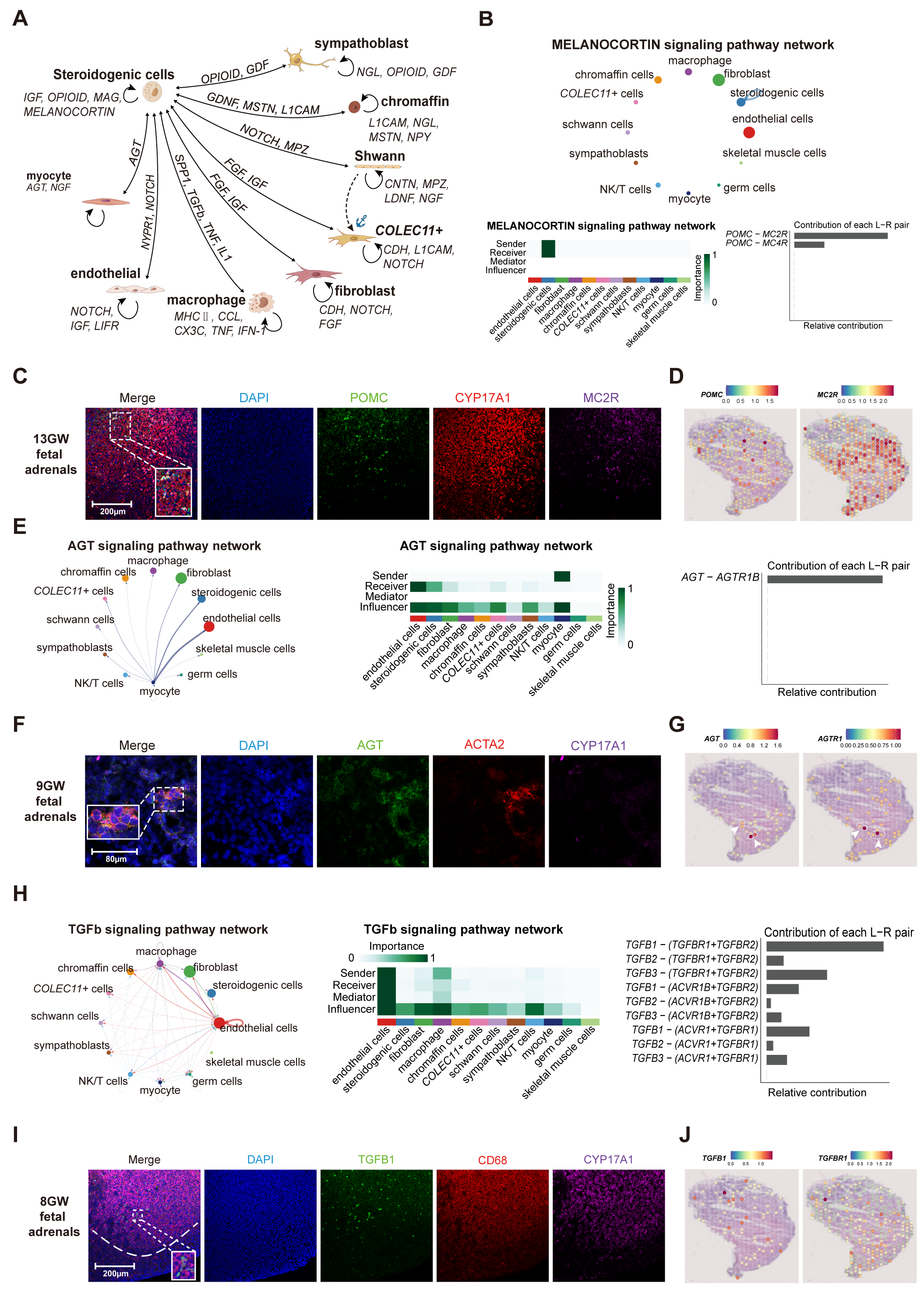

Supplement: Supplementary file 5 [file Image_5.tif]

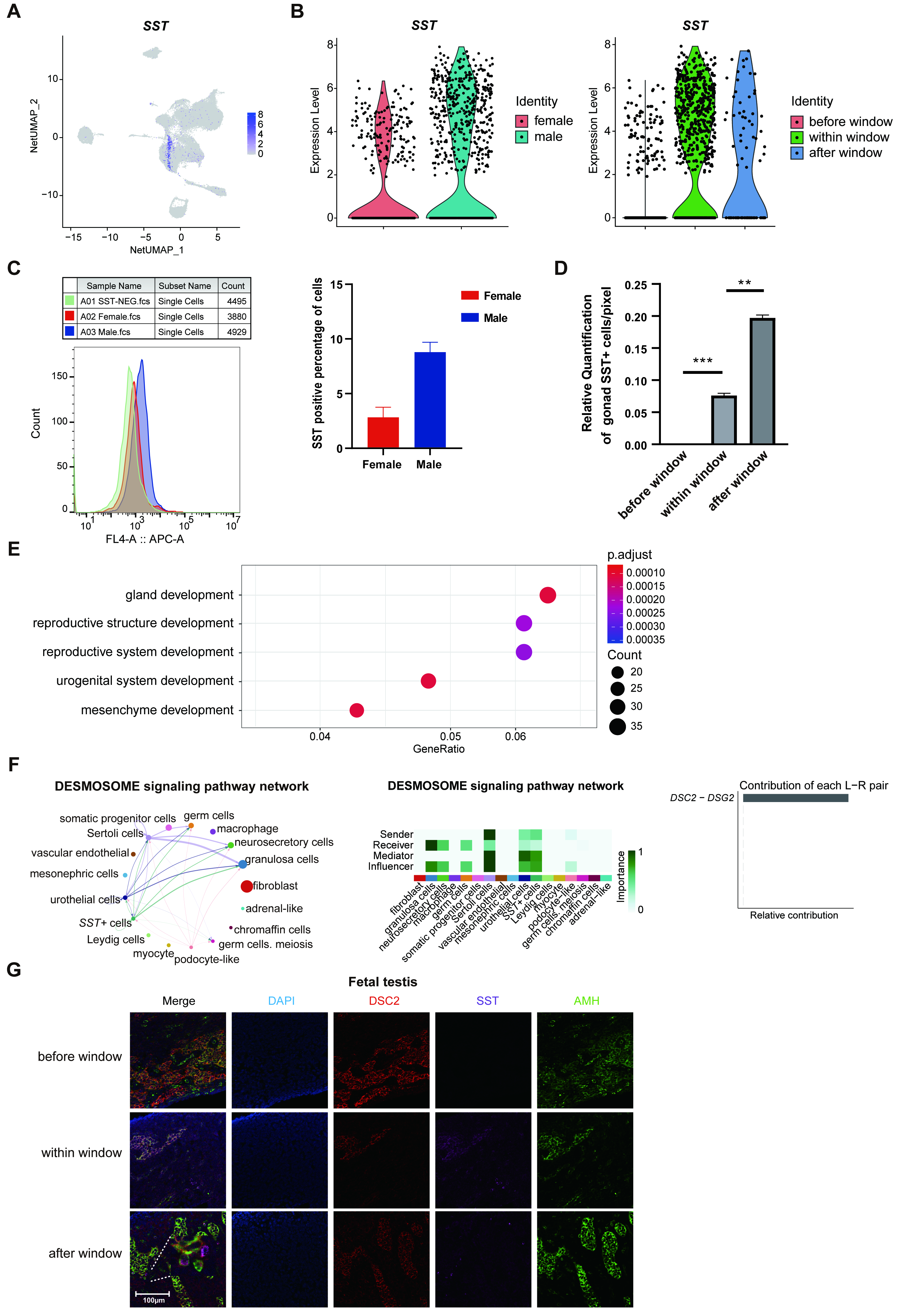

Supplement: Supplementary file 6 [file Image_6.tif]

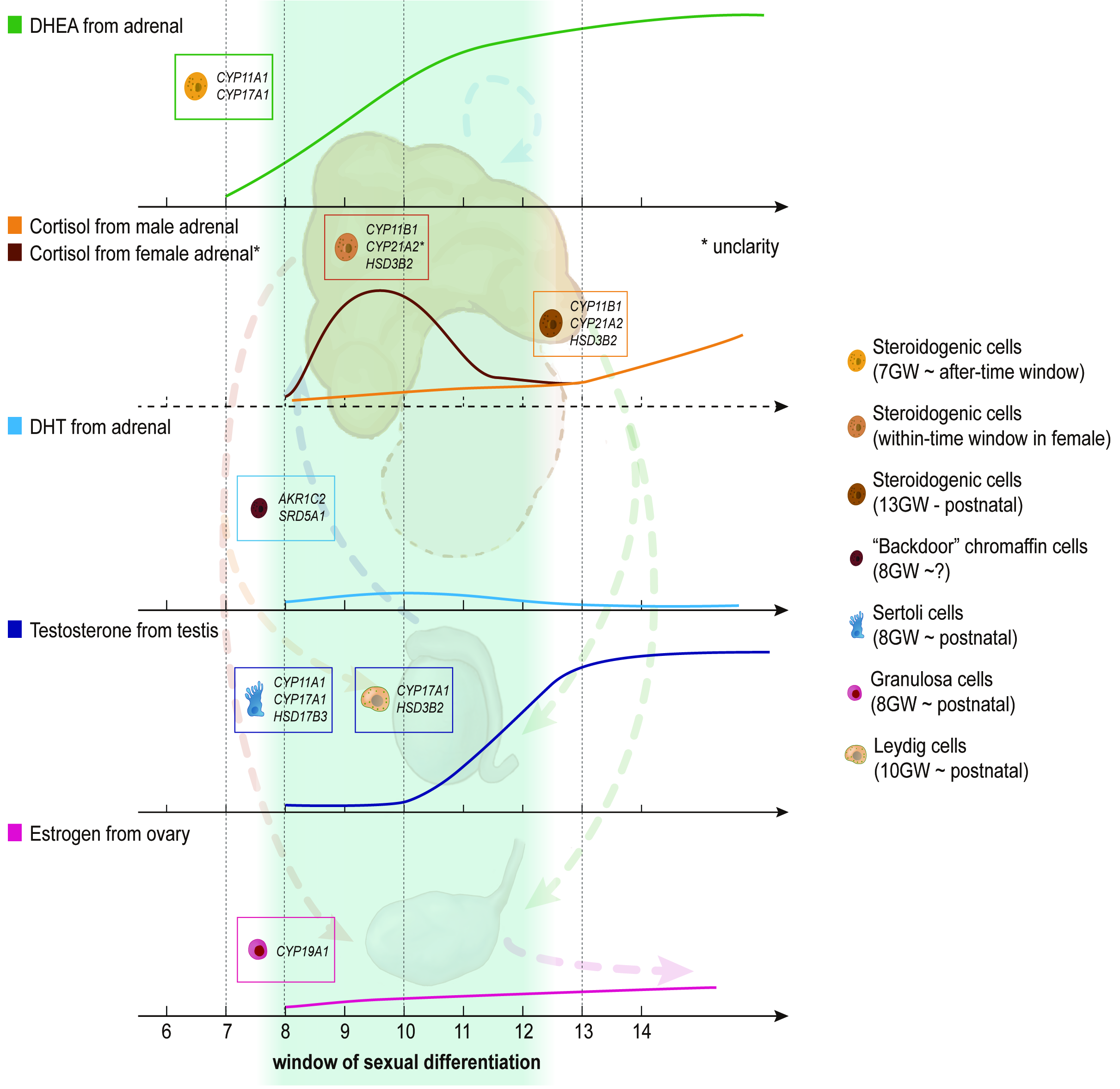

Supplement: Supplementary file 7 [file Image_7.tif]
