# Supplementary material for: A spatiotemporal steroidogenic regulatory network in human fetal adrenal glands and gonads
Source: Front Endocrinol (Lausanne). 2022 Nov 17;13:1036517. doi: 10.3389/fendo.2022.1036517 (PMC9713933; doi:10.3389/fendo.2022.1036517)
Supplement: Supplementary file 17 [file Table_10.docx]

| **Antibodies** | **SOURCE** | **IDENTIFIER** |
| --- | --- | --- |
| **rabbit anti-StAR** | **Novus** | **Cat# NBP1-33485; RRID:** [**AB_2197666**](https://scicrunch.org/resolver/RRID:%20AB_2197666) |
| **mouse anti-HSD3B2** | **Santa Cruz Biotechnology** | **Cat# sc-515120; RRID:** [**AB_2721058**](https://scicrunch.org/resolver/RRID:%20AB_2721058) |
| **goat anti-CYP17A1** | **Novus** | **Cat# NB100-2842; RRID:** [**AB_789512**](https://scicrunch.org/resolver/AB_789512) |
| **rabbit anti-NOV** | **Invitrogen** | **Cat# PA5-27893; RRID:** [**AB_2545369**](https://scicrunch.org/resolver/RRID:%20AB_2545369) |
| **mouse anti-CYP17A1** | **Santa Cruz Biotechnology** | **Cat# sc-374244; RRID:** [**AB_10988393**](https://scicrunch.org/resolver/AB_10988393) |
| **goat anti-MC2R** | **Novus** | **Cat# NB100-93419; RRID:** [**AB_1237169**](https://scicrunch.org/resolver/AB_1237169) |
| **mouse anti-Chromogranin A (LK2H10)** | **Invitrogen** | **Cat# MA5-13096; RRID:** [**AB_10987033**](https://scicrunch.org/resolver/AB_10987033) |
| **mouse anti-AKR1C2** | **Novus** | **Cat# NBP2-79775** |
| **goat anti-SRD5A1** | **Novus** | **Cat# NB100-1491; RRID:** [**AB_2255212**](https://scicrunch.org/resolver/RRID:%20AB_2255212) |
| **rabbit anti-CD5L** | **Novus** | **Cat# NBP1-76700; RRID:** [**AB_11020303**](https://scicrunch.org/resolver/AB_11020303) |
| **mouse anti-CD68** | **Invitrogen** | **Cat# 14-0688-82; RRID:** [**AB_11151139**](https://scicrunch.org/resolver/AB_11151139) |
| **rabbit anti-CHGA** | **Proteintech** | **Cat# 10529-1-AP; RRID:** [**AB_2081122**](https://scicrunch.org/resolver/RRID:%20AB_2081122) |
| **mouse anti-NPY** | **Invitrogen** | **Cat# ABS 028-08-02; RRID:** [**AB_1077304**](https://scicrunch.org/resolver/AB_1077304) |
| **rat anti-SST** | **Invitrogen** | **Cat# MA5-16987; RRID:** [**AB_2538460**](https://scicrunch.org/resolver/AB_2538460) |
| **rabbit anti-POMC** | **Bioss** | **Cat# bs-6942R** |
| **rabbit anti-TGFb1** | **Bioss** | **Cat# bs-0086R; RRID:** **[AB_10856457](https://scicrunch.org/resolver/AB_10856457)** |
| **rabbit anti-AGT** | **Novus** | **Cat# NBP1-30027SS** |
| **mouse anti-ACTA2** | **Invitrogen** | **Cat# MA1-06110; RRID:** [**AB_557419**](https://scicrunch.org/resolver/AB_557419) |
| **rabbit anti-AMH** | **Invitrogen** | **Cat# PA5-35851; RRID:** [**AB_2553161**](https://scicrunch.org/resolver/%20AB_2553161) |
| **mouse anti-DSC2** | **Invitrogen** | **Cat# 32-6200; RRID:** [**AB_2533090**](https://scicrunch.org/resolver/AB_2533090) |
| **Alexa Fluor 647-conjugated donkey anti-goat IgG** | **Abcam** | **Cat# ab150135; RRID:** [**AB_2687955**](https://scicrunch.org/resolver/AB_2687955) |
| **Alexa Fluor 594-conjugated donkey anti-mouse IgG** | **Abcam** | **Cat# ab150112; RRID:** [**AB_2813898**](https://scicrunch.org/resolver/AB_2813898) |
| **Alexa Fluor 488-conjugated donkey anti-rabbit IgG** | **Abcam** | **Cat# ab150061; RRID:** [**AB_2571722**](https://scicrunch.org/resolver/%20AB_2571722) |
| **Alexa Fluor 647-conjugated donkey anti-rat IgG** | **Abcam** | **Cat# ab150155; RRID:** [**AB_2813835**](https://scicrunch.org/resolver/AB_2813835) |
| **Alexa Fluor 488-conjugated donkey anti-mouse IgG** | **Abcam** | **Cat# ab150117; RRID:** [**AB_2688012**](https://scicrunch.org/resolver/AB_2688012) |
